# Historical Redlining, Social Determinants of Health, and Stroke Prevalence in Communities in New York City

**DOI:** 10.1001/jamanetworkopen.2023.5875

**Published:** 2023-04-05

**Authors:** Benjamin M. Jadow, Liangyuan Hu, Jungang Zou, Daniel Labovitz, Chinwe Ibeh, Bruce Ovbiagele, Charles Esenwa

**Affiliations:** 1 Department of Neurology, Montefiore Medical Center, Albert Einstein College of Medicine, Bronx, New York; 2 Department of Biostatistics and Epidemiology, Rutgers University, Piscataway, New Jersey; 3 Department of Biostatistics, Columbia University, New York, New York; 4 Department of Neurology, Columbia University, New York, New York; 5 Department of Neurology, University of California, San Francisco

## Abstract

**Importance:**

Historical redlining was a discriminatory housing policy that placed financial services beyond the reach of residents in inner-city communities. The extent of the impact of this discriminatory policy on contemporary health outcomes remains to be elucidated.

**Objective:**

To evaluate the associations among historical redlining, social determinants of health (SDOH), and contemporary community-level stroke prevalence in New York City.

**Design, Setting, and Participants:**

An ecological, retrospective, cross-sectional study was conducted using New York City data from January 1, 2014, to December 31, 2018. Data from the population-based sample were aggregated on the census tract level. Quantile regression analysis and a quantile regression forests machine learning model were used to determine the significance and overall weight of redlining in relation to other SDOH on stroke prevalence. Data were analyzed from November 5, 2021, to January 31, 2022.

**Exposures:**

Social determinants of health included race and ethnicity, median household income, poverty, low educational attainment, language barrier, uninsurance rate, social cohesion, and residence in an area with a shortage of health care professionals. Other covariates included median age and prevalence of diabetes, hypertension, smoking, and hyperlipidemia. Weighted scores for historical redlining (ie, the discriminatory housing policy in effect from 1934 to 1968) were computed using the mean proportion of original redlined territories overlapped on 2010 census tract boundaries in New York City.

**Main Outcomes and Measures:**

Stroke prevalence was collected from the Centers for Disease Control and Prevention 500 Cities Project for adults 18 years and older from 2014 to 2018.

**Results:**

A total of 2117 census tracts were included in the analysis. After adjusting for SDOH and other relevant covariates, the historical redlining score was independently associated with a higher community-level stroke prevalence (odds ratio [OR], 1.02 [95% CI, 1.02-1.05]; *P* < .001). Social determinants of health that were positively associated with stroke prevalence included educational attainment (OR, 1.01 [95% CI, 1.01-1.01]; *P* < .001), poverty (OR, 1.01 [95% CI, 1.01-1.01]; *P* < .001), language barrier (OR, 1.00 [95% CI, 1.00-1.00]; *P* < .001), and health care professionals shortage (OR, 1.02 [95% CI, 1.00-1.04]; *P* = .03).

**Conclusions and Relevance:**

This cross-sectional study found that historical redlining was associated with modern-day stroke prevalence in New York City independently of contemporary SDOH and community prevalence of some relevant cardiovascular risk factors.

## Key Points

**Question:**

Is the historical discriminatory housing policy known as redlining associated with modern-day stroke prevalence in New York City neighborhoods?

**Findings:**

In this cross-sectional study of 2117 census tracts in New York City, historical redlining was independently associated with community-level stroke risk beyond recognized social determinants of health.

**Meaning:**

Structural racism in the form of historical housing discrimination may be a factor in community stroke risk upstream of known social determinants of health.

## Teaser

This cross-sectional study examines whether redlining, as defined by the original designations of best, desirable, declining, and hazardous for investment, is associated with modern-day community level stroke disparities in the 2117 census tracts of New York City.

## Introduction

National incidence of stroke has declined steadily for the last half century, yet racial and ethnic stroke disparities continue to widen.^1,2^ While an increased burden of well-established cardiovascular risk factors likely accounts for much of the excess individual-level risk, social determinants of health (SDOH), which may include exposure to harmful social and environmental conditions, are increasingly recognized as contributing to disparate stroke outcomes, particularly in racial and ethnic minority communities.^3,4,5^

Additionally, aspects of structural racism may contribute to community stroke risk upstream of known SDOH. One example is the finding of a dose-dependent association between historical slave density and county-level modern-day stroke mortality in Black but not White communities in the US stroke belt, suggesting that a legacy of slavery, although many years upstream, continues to impact stroke risk in southeastern US communities.^6^ Similarly, starting in 1934, federally backed housing policies known as redlining disproportionately flagged many inner-city Black communities as hazardous, effectively divesting in their housing and economic development and excluding Black residents from home ownership. While the practice officially ended in 1968 with the passage of the Fair Housing Act, the socioeconomic impacts remain. Formerly redlined neighborhoods have less access to quality housing stock, transportation, schools, green space, sanitation services, and employment opportunities in the present day.^7^

In this population-level observational study, we sought to determine whether redlining, as defined by the original designations of best, desirable, declining, and hazardous for investment, are associated with modern-day community level stroke disparities in the 2117 census tracts of New York City. We also studied the role of SDOH in formerly redlined communities and hypothesized that there would be a residual association between historical redlining score (HRS) and stroke prevalence, even after controlling for known SDOH. Finally, we considered the independent role of race and/or ethnicity on stroke risk after controlling for classic cardiovascular risk factors, SDOH, and HRS.

## Methods

### Study Population and Design

This was an ecological cross-sectional study of the 2117 census tracts in New York City. This study was approved by the Institutional Review Board of the Montefiore Medical Center. The study followed the Strengthening the Reporting of Observational Studies in Epidemiology (STROBE) reporting guideline.

A total of 8 SDOH were selected in accordance with the framework used in previously published studies to reflect components of the Healthy People 2020 Framework.^8^ The SDOH were collected from the 2014-2018 American Community Survey and included Black race and/or Hispanic ethnicity, median household income, poverty, low educational attainment, language barrier, and uninsurance rate. Social cohesion was collected from the New York City Department of Health’s Community Health Survey conducted from January 1, 2014, to December 31, 2018. All data in this survey were self-reported, including race and ethnicity, and were transformed onto 2020 census tract boundaries. Residence in an area with a shortage of health care professionals was defined by the Health Resources and Services Administration, and we used designations from 2018 (eTable 1 in Supplement 1). Each SDOH variable was chosen to reflect a specific element of the Healthy People Framework. Social and community variables were Black race and/or Hispanic ethnicity, social cohesion, and language barrier. Education was represented by low educational attainment. Economic stability was represented by median household income. Neighborhood and/or environment was represented by poverty, and health and health care was represented by uninsurance rate and shortage of health care professionals.

Weighted HRS based on the 4 original Homeowner’s Loan Corporation residential security grades (best, desirable, declining, and hazardous for investment) overlapped on the 2010 census tract boundaries of New York City were obtained from the Inter-University Consortium for Political and Social Research at the University of Michigan.^9^ Census tract HRS were computed using the mean proportion of original redlined territory contained within each census tract, similar to methods used in prior studies.^10,11^ Stroke prevalence, including both ischemic and hemorrhagic, and relevant covariates including median age and prevalence of diabetes, hypertension, smoking, and hyperlipidemia were collected for adults 18 years or older at the census tract level from the Centers for Disease Control and Prevention 500 Cities Project (Figure). We used prevalence data from 2018 to reflect the final year of American Community Survey data collection. Median age was used because it is less prone to outliers than mean age, and covariates were selected to represent the most upstream medical factors that put patients at risk for stroke.

**Figure.**
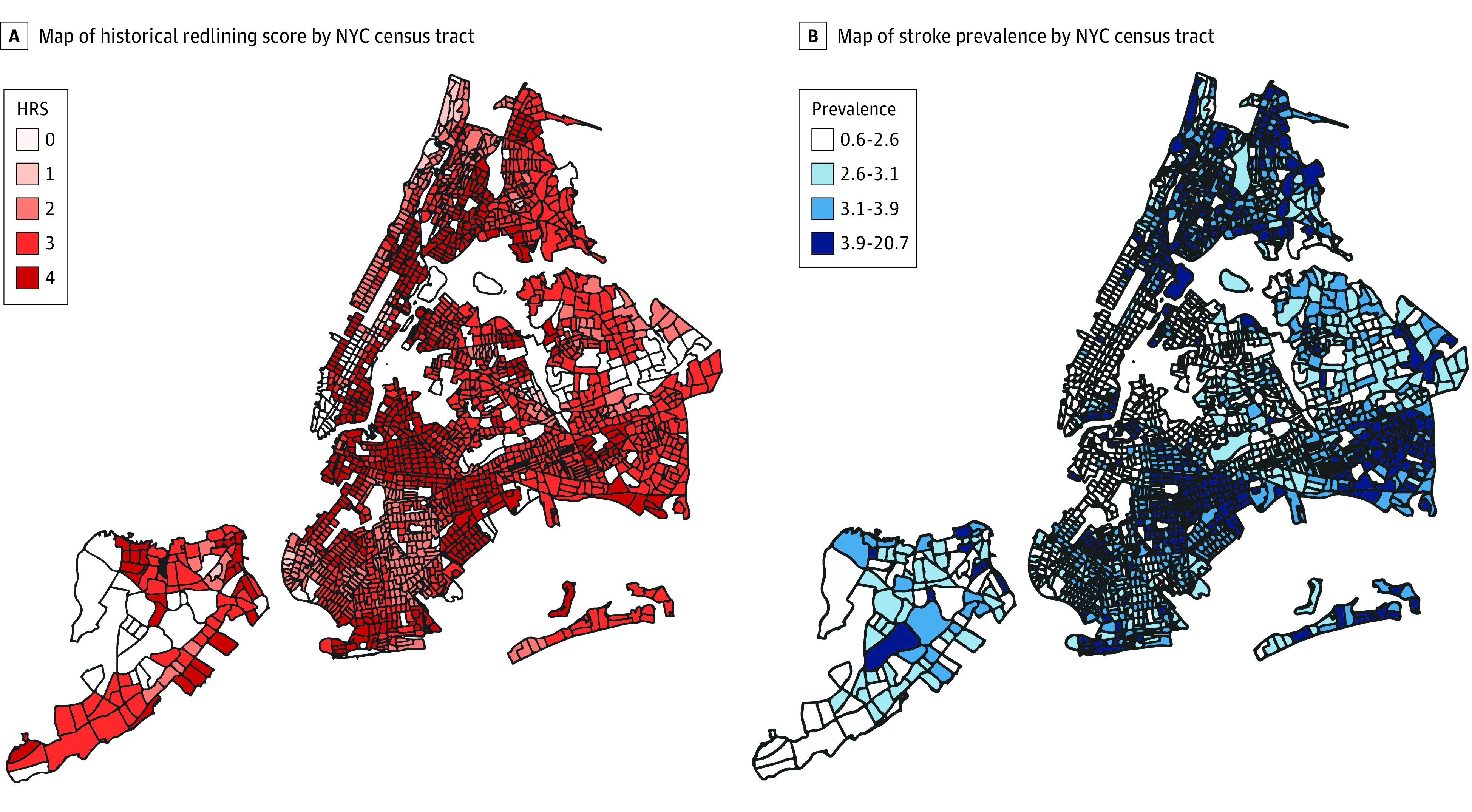
Maps of Historical Redlining Score (HRS) and Stroke Prevalence by New York City (NYC) Census Tracts

### Statistical Analysis

Data were analyzed from November 5, 2021, to January 31, 2022. Univariate analyses were calculated for each SDOH using quantile regression at the 50th percentile, represented by the unadjusted column in Table 1. Quantile regression models were calculated for each SDOH separately, adjusting for traditional stroke risk factors, represented by the adjusted column in Table 1. Variables with a threshold of 2-sided *P* < .10 were retained for further analysis in stepwise quantile regression models also at the 50th percentile. Quantile regression was used because it gives a fuller picture about the association of the exposure with the entire distribution of the outcome. Moreover, it does not require the normality assumption for the outcome. Stepwise regression modeling was used to determine the extent to which SDOH variables might serve as moderators between redlining and stroke (eTable 2 in Supplement 1). Regression coefficients and odds ratios (ORs) represent changes in the median stroke prevalence for a 1-unit change in HRS. A machine learning–based quantile regression forest (QRF) was conducted to assess the overall relevance of redlining relative to other covariates, including SDOH, with community-level stroke prevalence.^12^ This analysis weighs all variables in the study and ranks them based on the extent of their influence on the outcome (eFigure in Supplement 1). Compared with the parametric quantile regression model, the QRF offers a substantial level of modeling flexibility to capture the true covariate-outcome associations, based on which we compute the relative importance scores of the covariates. Diabetes prevalence and smoking prevalence were removed because of an absolute correlation higher than 0.75 with hypertension and poverty. After the correlation reduction, there were a total of 13 features. All analyses were conducted using SPSS Statistics, version 28.0.0.0 (IBM Corporation), and R, version 3.6.1 (quantregForest package; R Project for Statistical Computing). Statistical significance was established at *P* < .01.

**Table 1.** Associations Between SDOH and Stroke Prevalence

SDOH	Unadjusted		Adjusted^a^	
	OR (95% CI)	*P* value	OR (95% CI)	*P* value
Median household income	1.00 (1.00-1.00)	<.001^b^	1.00 (1.00-1.00)	NA
Educational attainment	1.04 (1.04-1.05)	<.001^b^	1.01 (1.01-1.01)	<.001^b^
Poverty	1.05 (1.04-1.05)	<.001^b^	1.01 (1.01-1.01)	<.001^b^
Black race and/or Hispanic ethnicity	1.02 (1.02-1.02)	<.001^b^	1.00 (1.00-1.00)	<.001^b^
Language barrier	1.00 (.99-1.00)	>.99	1.00 (1.00-1.00)	<.001^b^
Social cohesion	0.97 (0.96-0.98)	<.001^b^	1.00 (1.00-1.00)	<.001^b^
HRS	1.24 (1.16-1.32)	<.001^b^	1.02 (1.02-1.05)	<.001^b^
Uninsurance rate	1.02 (1.01-1.03)	<.001^b^	1.00 (1.00-1.00)	<.001^b^
Health care professional shortage	1.65 (1.48-1.84)	<.001^b^	1.02 (1.00-1.05)	.03

Abbreviations: HRS, historical redlining score; OR, odds ratio; SDOH, social determinants of health.

^a^ Adjusted for median age and prevalence of diabetes, smoking, hypertension, and hypercholesterolemia by census tract.

^b^ Significant at *P* < .01.

## Results

In unadjusted analysis of the 2117 census tracts in New York City, 7 of the 8 studied SDOH were found to be associated with community-level stroke prevalence. These included low levels of educational attainment (OR, 1.04 [95% CI, 1.04-1.05]; *P* < .001), poverty (OR, 1.05 [95% CI, 1.04-1.05]; *P* < .001), Black race and/or Hispanic ethnicity (OR, 1.02 [95% CI, 1.02-1.02]; *P* < .001), uninsurance rate (OR, 1.02 [95% CI, 1.01-1.03]; *P* < .001), and health care professional shortage (OR, 1.65 [95% CI, 1.48-1.84]; *P* < .001), all of which carried a positive association, and household income (OR, 1.00 [95% CI, 1.00-1.00]; *P* < .001) and social cohesion (OR, 0.97 [95% CI, 0.96-0.98]; *P* < .001), which were negatively associated with community-level stroke prevalence. The HRS carried a positive association with community-level stroke prevalence in unadjusted modeling (OR, 1.24 [95% CI, 1.16-1.32]; *P* < .001) **(**Table 1).

After adjusting for age and prevalence of diabetes, smoking, hypertension, and hypercholesterolemia, educational attainment (OR, 1.01 [95% CI, 1.01-1.01]; *P* < .001), poverty (OR, 1.01 [95% CI, 1.01-1.01]; *P* < .001), language barrier (OR. 1.00 [95% CI, 1.00-1.00]; *P* < .001), health care professional shortage (OR, 1.02 [95% CI, 1.00-1.05]; *P* < .001), and HRS (OR, 1.02 [95% CI, 1.02-1.05]; *P* < .001) maintained a positive association with stroke prevalence. Black race and/or Hispanic ethnicity (OR, 1.00 [95% CI, 1.00-1.00]; *P* < .001) and uninsurance rate (OR, 1.00 [95% CI, 0.99-1.00]; *P* < .001) were negatively associated with stroke (Table 1).

In a stepwise logistic regression model, increased HRS was independently associated with community-level stroke prevalence (OR, 1.04 [95% CI, 1.02-1.05]; *P* < .001) after adjusting for common cardiovascular risk factors and SDOH (model 5 in Table 2). The QRF machine learning model ranked the variables of interest in the following order from most to least important by association with stroke prevalence: household income, educational attainment, Black race and/or Hispanic ethnicity, language barrier, social cohesion score, uninsurance rate, HRS, and health care professional shortage.

**Table 2.** Stepwise Regression Model of Redlining, SDOH, and Stroke Prevalence

Covariate	Model 1		Model 2^a^		Model 3^b^		Model 4^c^		Model 5^d^	
	OR (95% CI)	*P* value	OR (95% CI)	*P* value	OR (95% CI)	*P* value	OR (95% CI)	*P* value	OR (95% CI)	*P* value
HRS	1.35 (1.16-1.32)	<.001^e^	1.02 (1.02-1.05)	<.001^e^	1.02 (1.01-1.04)	<.001^e^	1.04 (1.03-1.05)	<.001^e^	1.04 (1.02-1.05)	<.001^e^
Median age	NA	NA	1.00 (1.00-1.00)	.04	1.00 (1.00-1.00)	.03	0.99 (0.99-0.99)	<.001^e^	0.99 (0.99-1.00)	<.001
Hypertension prevalence	NA	NA	1.15 (1.15-1.15)	<.001^e^	1.15 (1.14-1.15)	<.001^e^	1.16 (1.16-1.17)	<.001^e^	1.16 (1.16-1.17)	<.001^e^
Diabetes prevalence	NA	NA	1.01 (1.00-1.01)	.052	1.01 (1.01-1.02)	<.001^e^	1.03 (1.02-1.04)	<.001^e^	1.04 (1.02-1.05)	<.001
Smoking prevalence	NA	NA	1.05 (1.04-1.05)	<.001^e^	1.05 (1.04-1.05)	<.001^e^	1.02 (1.01-1.02)	<.001^e^	1.00 (0.99-1.00)	.31
Hyperlipidemia prevalence	NA	NA	0.99 (0.99-1.00)	<.001^e^	0.99 (0.99-0.99)	<.001^e^	0.98 (0.98-0.99)	<.001^e^	0.99 (0.98-0.99)	<.001
Uninsurance rate	NA	NA	NA	NA	1.00 (0.99-1.00)	<.001^e^	1.00 (0.99-1.00)	<.001^e^	1.00 (1.00-1.00)	.06
Social cohesion	NA	NA	NA	NA	1.00 (1.00-1.00)	.005^e^	1.00 (1.00-1.00)	.34	1.00 (1.00-1.01)	.002
Black race and/or Hispanic ethnicity	NA	NA	NA	NA	NA	NA	1.00 (1.00-1.00)	<.001^e^	1.00 (1.00-1.00)	<.001^e^
Educational attainment	NA	NA	NA	NA	NA	NA	1.01 (1.01-1.01)	<.001^e^	1.01 (1.01-1.01)	<.001^e^
Language barrier	NA	NA	NA	NA	NA	NA	NA	NA	1.00 (1.00-1.00)	.002^e^
Health care professional shortage	NA	NA	NA	NA	NA	NA	NA	NA	1.04 (1.01-1.06)	.004^e^
Poverty	NA	NA	NA	NA	NA	NA	NA	NA	1.01 (1.01-1.01)	<.001^e^

Abbreviations: HRS, historical redlining score; NA, not applicable; OR, odds ratio; SDOH, social determinants of health.

^a^ Adjusted for age and prevalence of diabetes, hypertension, hyperlipidemia, and smoking.

^b^ Adjusted for covariates in model 2, uninsurance rate, and social cohesion.

^c^ Adjusted for covariates in model 3, Black race and/or Hispanic ethnicity, and educational attainment.

^d^ Adjusted for covariates in model 4, language barrier, health care professional shortage, and poverty.

^e^ Significant at *P* < .01.

## Discussion

In 1934, the Federal Housing Administration incorporated redlining into its mortgage underwriting criteria as a form of credit rationing to curb a national decline in the housing market. The practice of redlining rated many inner-city neighborhoods in at least 239 US cities as hazardous based on racial composition, poverty level, housing stock, and stability of housing value. In this ecological cross-sectional study, we found an association between New York City communities historically disadvantaged by poor HRS and modern-day community-level stroke prevalence. While HRS carried less importance than some other SDOH, the observed association of HRS with stroke prevalence remained even after controlling for common cardiovascular risk factors and SDOH. While further research is needed, these results suggest that there may be residual effects of HRS on community stroke risk in certain New York City communities that are additive to classic SDOH defined by the Healthy People Framework. Similar to residual effects on community stroke risk stemming from a legacy of slavery in the southeastern US, redlining may be yet another example of historical structural racism with enduring effects on community-level cardiovascular health and specifically stroke prevalence, disproportionately affecting racial and ethnic minority communities.

The mechanisms by which HRS may be contributing across generations to current neighborhood-level stroke risk is likely complex. A potentially underappreciated association may be a high burden of chronic environmental stress both on the individual and community level.^13,14,15,16^ It is plausible that constant adverse circumstances associated with living in a historically disinvested community would lead to a lifetime and perhaps generational burden of hard-to-measure physical, psychological, and financial stress. Further, measured or unmeasured SDOH are likely compounded by exposure to other associated environmental stressors like community violence, increased rates of mental illness, food insecurity, internalized discrimination, and personally mediated racism, all of which are cumulative over a lifespan.^17,18,19,20,21,22,23^ It is also plausible that HRS created the conditions for the hazardous SDOH examined in this study and that the SDOH themselves served as the link between public policy and contemporary health.

Interestingly, a higher community prevalence of Black race and/or Hispanic ethnicity was negatively associated with neighborhood stroke prevalence after fully adjusting for relevant risk factors, SDOH, and HRS. While surprising, this finding is consistent with our understanding of the role of race and ethnicity as an identifier of social position rather than a biological risk factor. A similar interplay among social standing, race, and stroke has previously been documented and is well established in other chronic diseases with disparate outcomes related to race and ethnicity.^24,25^ The present study adds to the literature by solidifying the association of historical redlining and modern stroke prevalence and is in line with other recent studies showing associations between HRS and community burden of cardiovascular disease.^10,11,23,26,27^

### Strengths and Limitations

This study is strengthened by the large sample size and use of survey data from the largest and perhaps most diverse city in the US. A major limitation to this study is that variables were observed on the population level, thus limiting inference to individuals. Population level studies also cannot necessarily draw conclusions about the direction of causality. However, the ecological design is necessary when studying the effects of historical practices on entire communities, as obtaining data on individuals across multiple generations in New York City is not feasible. Additionally, the reliance on self-reported variables may introduce recall bias and limit generalizability of the findings. The use of census tracts as the main unit of the analysis has the potential to introduce biases including spatial dependency, spillover, and the modifiable area unit problem. It also does not incorporate migration of individuals across census tracts. Additionally, the use of prevalence as the primary outcome excludes individuals who had a stroke and died or who had a stroke and migrated. Ascertainment of exposure to redlining presents many challenges in this field of study. Our study follows the precedent set by others in the field; however, future studies may consider alternative methods that have the capacity to draw conclusions about individuals.

## Conclusions

Beyond previously recognized SDOH and relevant cardiovascular risk factors, the findings of this cross-sectional study suggest that housing discrimination in the form historical redlining was associated with community stroke prevalence in New York City communities. Further research is needed to determine whether and through what pathways historical redlining may be contributing to community stroke risk in certain classically underserved inner-city communities.

## Notes

Supplement 1. **eTable 1.** Variable Definitions **eTable 2.** Stepwise Regression Model With Regression Coefficients **eFigure.** Description of Quantile Regression Forests Machine Learning Model

Supplement 2.
Data Sharing Statement
