# Supplementary material for: Historical Redlining, Social Determinants of Health, and Stroke Prevalence in Communities in New York City
Source: JAMA Netw Open. 2023 Apr 5;6(4):e235875. doi: 10.1001/jamanetworkopen.2023.5875 (PMC10077098; doi:10.1001/jamanetworkopen.2023.5875)
Supplement: Supplement 1. — eTable 1. Variable Definitions eTable 2. Stepwise Regression Model With Regression Coefficients eFigure. Description of Quantile Regression Forests Machine Learning Model [file jamanetwopen-e235875-s001.pdf]

Supplementary Online Content

Jadow BM, Hu L, Zou J, et al. Historical redlining, social determinants of health, and stroke prevalence in communities in New York City. *JAMA Netw Open*. 2023;6(4):e235875. doi:10.1001/jamanetworkopen.2023.5875

eTable 1. Variable Definitions

eTable 2. Stepwise Regression Model With Regression Coefficients

eFigure. Description of Quantile Regression Forests Machine Learning Model

This supplementary material has been provided by the authors to give readers additional information about their work.

© 2023 Jadow BM et al. *JAMA Network Open*.

eTable 1. Variable Definitions

Variable	Description	Source
Median Household Income	Median income reported for each household	ACS 2014-2018
Poverty	Percentage of individuals whose household income was below poverty level for the previous 12 months	ACS 2014-108
Low educational attainment	Percentage of individuals over the age of 25 who had completed less than high school degree	ACS 2014-2018
Black race/Hispanic ethnicity	Percentage of individuals who identify as Black or Hispanic	ACS 2014-2018
Language barrier	Percentage of individuals who speak English "less than very well"	ACS 2014-2018
Uninsurance rate	Percentage of individuals without health insurance	ACS 2014-2018
Social cohesion	Percentage of individuals who report that people in their neighborhood are willing to help their neighbors	Community Health Survey
Health care shortage	Was the census tract designated as a health care shortage area, yes or no	Health resources and services administration
Redlining score	Weighted historic redlining scores were calculated for each census tract	Inter-university Consortium for Political and Social Research (ICPSR)

© 2023 Jadow BM et al. *JAMA Network Open*.

eTable 2. Stepwise Regression Model With Regression Coefficients

OR<br>(95%CI)<br>p-value	Model 1 (unadjusted)	Model 2 (Adjusted for age, diabetes, hypertension, hyperlipidemia, smoking)	Model 3 (adjusted for model 2 + uninsurance rate + social cohesion	Model 4 (adjusted for model 3 + Black/Hispanic + educational attainment)	Model 5 (adjusted for model 4 + language barrier + health care shortage + poverty)
Redlining score	0.213<br>(0.145, 0.280)<br><.001*	0.023<br>(0.016, 0.053)<br><.001*	0.022<br>(0.010, 0.035)<br><.001*	0.038<br>(0.025, 0.052)<br><.001*	0.036<br>(0.022, 0.052)<br><.001*
Median age	-	-0.002<br>(-0.004, 0.000)<br>.038*	-0.002<br>(-0.004, 0.000)<br>.033*	-0.010<br>(-0.012, -0.008)<br><.001*	-0.007<br>(-0.009, -0.005)<br><.001
Hypertension	-	0.140<br>(0.136, 0.143)<br><.001*	0.137<br>(0.133, 0.140)<br><.001*	0.152<br>(0.148, 0.156)<br><.001*	0.151<br>(0.146, 0.156)<br><.001*
Diabetes	-	0.006<br>(0.000, 0.012)<br>.052	0.013<br>(0.006, 0.019)<br><.001*	0.026<br>(0.018, 0.035)<br><.001*	0.034<br>(0.024, 0.044)<br><.001
Smoking	-	0.045<br>(0.041, 0.049)<br><.001*	0.046<br>(0.042, 0.048)<br><.001*	0.017<br>(1.013, 1.022)<br><.001*	-0.003<br>(-0.008, 0.003)<br>.307
Hyperlipidemia	-	-0.008<br>(-0.011, -0.005)<br><.001*	-0.009<br>(-0.012, -0.006)<br><.001*	-0.016<br>(-0.019, -0.013)<br><.001*	-0.015<br>(-0.018, -0.002)<br><.001
Uninsurance rate	-	-	-0.004<br>(-0.006, -0.002)<br><.001*	-0.005<br>(-0.007, -0.003)<br><.001*	-0.002<br>(-0.004,0.000)<br>.061
Social Cohesion	-	-	-0.002<br>(-0.004, -0.001)<br>.005*	-0.001<br>(-0.002, 0.001)<br>.342	0.003<br>(0.001, 0.005)<br>.002
Black race and Hispanic ethnicity	-	-	-	-0.004<br>(-0.004, -0.003)<br><.001*	-.0.004<br>(-0.005, -0.004)<br><.001*
Educational Attainment	-	-	-	-0.006<br>(0.004, 0.007)<br><.001*	0.007<br>(0.005, 0.009)<br><.001
Language Barrier	-	-	-	-	-0.002<br>(-0.003, -0.001)<br>.002
Health care shortage	-	-	-	-	0.035<br>(0.011, 0.060)<br>.004
Poverty	-	-	-	-	1.009<br>(0.008, 0.010)<br><.001

© 2023 Jadow BM et al. *JAMA Network Open*.

eFigure. Description of Quantile Regression Forests Machine Learning Model

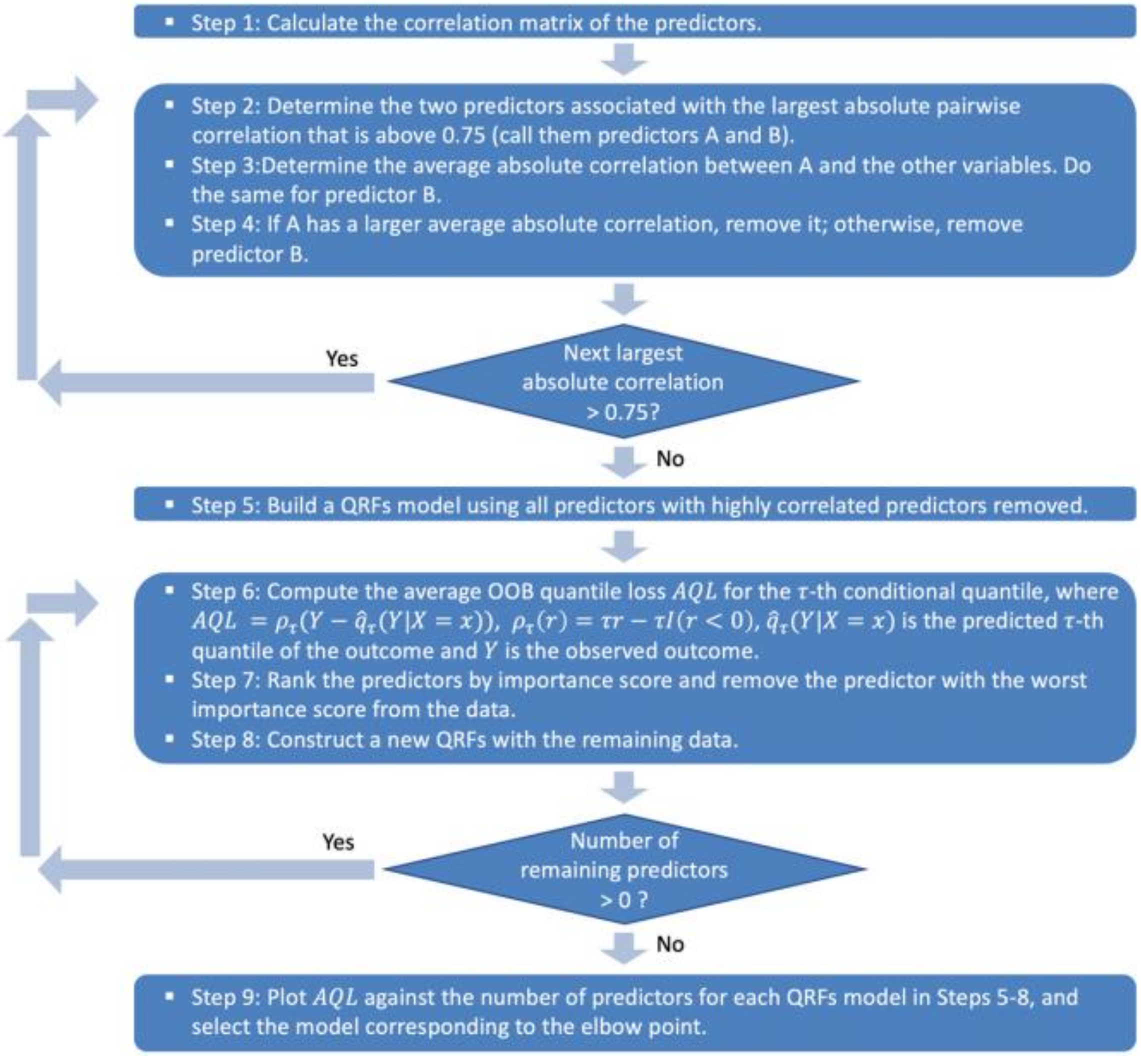

© 2023 Jadow BM et al. *JAMA Network Open*.
