# Supplementary material for: Historical Redlining, Social Determinants of Health, and Stroke Prevalence in Communities in New York City
Source: JAMA Netw Open. 2023 Apr 5;6(4):e235875. doi: 10.1001/jamanetworkopen.2023.5875 (PMC10077098; doi:10.1001/jamanetworkopen.2023.5875)
Supplement: Supplement 2. — Data Sharing Statement [file jamanetwopen-e235875-s002.pdf]

Data Sharing Statement

Jadow. Historical Redlining, Social Determinants of Health, and Stroke Prevalence in Communities in New York City. *JAMA Netw Open*. Published April 05, 2023. doi:10.1001/jamanetworkopen.2023.5875

Data
Data available: Yes
Data types: Data (not involving human participants)
How to access data: benjamin.jadow@einsteinmed.edu
When available: With publication

Supporting Documents
Document types: None

Additional Information
Who can access the data: Anyone requesting the data
Types of analyses: For any purpose
Mechanisms of data availability: With investigator support
